# Pathogenic Variant Spectrum in Breast Cancer Risk Genes in Finnish Patients

**DOI:** 10.3390/cancers14246158

**Published:** 2022-12-14

**Authors:** Anna K. Nurmi, Maija Suvanto, Joe Dennis, Kristiina Aittomäki, Carl Blomqvist, Heli Nevanlinna

**Affiliations:** 1 Department of Obstetrics and Gynecology, University of Helsinki and Helsinki University Hospital, 00029 Helsinki, Finland; 2 Centre for Cancer Genetic Epidemiology, Department of Public Health and Primary Care, University of Cambridge, Cambridge CB1 8RN, UK; 3 Department of Clinical Genetics, University of Helsinki and Helsinki University Hospital, 00029 Helsinki, Finland; 4 Department of Oncology, University of Helsinki and Helsinki University Hospital, 00029 Helsinki, Finland

**Keywords:** breast cancer, pathogenic variant, gene-panel sequencing, copy number variant, *BRCA1*, *BRCA2*, moderate-risk gene

## Abstract

**Simple Summary:**

The Finnish population has evolved through multiple reductions in the population size, which have caused decreased genetic diversity in the population. This may affect the risk variant spectrum in diseases such as breast cancer (BC) so that a few variants may cover most of the pathogenic variation found in the risk genes. A dozen recurrent pathogenic variants have been identified in the moderate-risk BC susceptibility genes in Finnish BC patients. To evaluate the spectrum and frequency of the risk variants more comprehensively, we have, here, studied all variants in 1769 patients and copy number changes in 1511 patients both in the moderate-risk genes as well as in the high-risk *BRCA1* and *BRCA2* genes. While the overall pathogenic variant frequency was comparable to other populations, just a few variants accounted for most of the pathogenic burden in the risk genes. These results could be utilized in population screening strategies in Finland.

**Abstract:**

Recurrent pathogenic variants have been detected in several breast and ovarian cancer (BC/OC) risk genes in the Finnish population. We conducted a gene-panel sequencing and copy number variant (CNV) analysis to define a more comprehensive spectrum of pathogenic variants in *BRCA1*, *BRCA2*, *PALB2*, *CHEK2*, *ATM*, *BARD1*, *RAD51C*, *RAD51D*, *BRIP1*, and *FANCM* genes in Finnish BC patients. The combined frequency of pathogenic variants in the *BRCA1/2* genes was 1.8% in 1356 unselected patients, whereas variants in the other genes were detected altogether in 8.3% of 1356 unselected patients and in 12.9% of 699 familial patients. CNVs were detected in 0.3% of both 1137 unselected and 612 familial patients. A few variants covered most of the pathogenic burden in the studied genes. Of the *BRCA1/2* carriers, 70.8% had 1 of 10 recurrent variants. In the other genes combined, 92.1% of the carrier patients had at least 1 of 11 recurrent variants. In particular, *PALB2* c.1592delT and *CHEK2* c.1100delC accounted for 88.9% and 82.9%, respectively, of the pathogenic variation in each gene. Our results highlight the importance of founder variants in the BC risk genes in the Finnish population and could be used in the designing of population screening for the risk variants.

## 1. Introduction

With a lifetime risk of 13%, breast cancer (BC) is the most frequently-diagnosed cancer in Finnish women [1]. Several high- or moderate-penetrance genes have been determined to be clinically valid for the prediction of BC risk [2,3]. Pathogenic variants in *BRCA1* lead to high cumulative lifetime risks of BC and ovarian cancer (OC) (72% and 44%), respectively, when the estimates are 69% and 17% for *BRCA2* [4]. While BC risk associated with deleterious *PALB2* variants exceeds the threshold for a high-risk gene (OR > 5), variants in *CHEK2*, *ATM*, *BARD1*, *RAD51C*, and *RAD51D* lead to a moderate increase in the BC risk (OR 2–3) [3,5]. Additionally, *RAD51C*, *RAD51D*, and *BRIP1* are validated OC susceptibility genes, predisposing a carrier to a high risk of OC [6,7].

The BC risk effects may be stronger for specific breast tumor subtypes. Pathogenic *ATM* and *CHEK2* variants predispose carriers to a higher risk of estrogen receptor (ER)-positive BC than ER-negative BC, while variants in *PALB2* and the other moderate-risk genes increase, especially, the risk of ER-negative and triple-negative BC [3,5,8]. *FANCM* is a prospective moderate-risk gene which is associated with ER-negative and triple-negative BC; however, the exact, variant-specific risks remain to be established [3,9,10]. Furthermore, a great number of identified common variants with a small individual effect on the BC predisposition [11,12], combined into a polygenic risk score (PRS), have recently been shown to modify the risk caused by high- and moderate-penetrance variants [13,14,15].

In addition to single-nucleotide and short insertion-deletion polymorphisms (SNPs and indels), copy-number variants (CNVs) are prevalent in the human genome and can be pathogenic [16]. Exon-level deletions and duplications have been identified in multiple BC predisposition genes, for instance in *BRCA1* and *CHEK2* [17,18,19]. CNVs are harder to detect accurately than SNPs, and investigations of their contribution to BC risk are ongoing [20].

The Finnish population has evolved through several well-documented population bottleneck events combined with geographical and cultural isolation, making its genetic constitution distinctive [21,22]. Less genetic variation but more low-frequency loss-of-function (LoF) variants have been reported in Finns compared to non-Finnish Europeans [21]. Furthermore, a single variant covers the majority of pathogenic variation in most of the monogenic Finnish heritage diseases [22]; however, the founder effect can also affect more common, polygenic diseases such as BC.

Pathogenic *BRCA1/2* variants have been observed in 21% of Finnish BC families and recurrent founder variants in 1.8% of unselected BC patients [23,24]. Just 12 recurrent pathogenic variants have been detected in the *PALB2*, *CHEK2*, *ATM*, *RAD51C*, *RAD51D*, and *FANCM* genes in Finnish BC patients; we have estimated the overall frequency of these variants to be 7.5% in unselected and 13.3% in familial BC patients [25]. However, the full spectrum and frequency of pathogenic variants is not yet known. In this study, we aim to analyze the prevalence of pathogenic LoF and missense variants more comprehensively in the moderate-risk genes as well as in the *BRCA1/2* genes in Finnish BC patients.

## 2. Materials and Methods

### 2.1. Patient and Control Series

The patient series consisted of 1769 female BC patients from the Helsinki region in Finland. The unselected BC series comprised 1356 patients with first diagnosed invasive tumor, recruited without selection for family history of the disease or age of diagnosis in the Helsinki University Hospital at the Department of Oncology in 1997–1998 and 2000 [24,26] and at the Department of Surgery in 2001–2004 [27]. The familial BC series included 286 patients collected among the unselected series and 413 additional BC patients recruited at the Department of Oncology and the Department of Clinical Genetics until 2015 [27,28,29], totaling 699 familial index cases. Of them, 340 index patients had a family history of at least three individuals affected with BC or OC among first- or second-degree relatives (including the proband) and 359 had one affected first-degree relative. The carriers of pathogenic *BRCA1/2* variants had been excluded from the familial series. The population controls comprised 1112 female blood donors from the same geographical region. Genotyping was carried out using genomic DNA extracted from peripheral blood samples.

### 2.2. Gene Selection

We focused on the validated BC and OC risk genes *BRCA1*, *BRCA2*, *PALB2*, *CHEK2*, *ATM*, *BARD1*, *RAD51C*, *RAD51D*, and *BRIP1*, and the putative moderate-risk gene *FANCM*, and analyzed the SNPs and short indels along with the CNVs detected in these genes in our patient and control series. In this study, we analyzed *PALB2* among the moderate-risk genes.

### 2.3. Gene-Panel Sequencing

The DNA samples were sequenced as part of a Breast Cancer Association Consortium’s (BCAC) panel analysis of 34 confirmed and suspected BC susceptibility genes. The genotyping and variant annotation process has been described by Dorling et al. [3].

### 2.4. Single-Nucleotide Variants and Short Indels in Moderate-Risk Genes

To cover a comprehensive spectrum of damaging variants in the moderate-risk genes in the studied Finnish BC patients, we examined all pathogenic variants as well as variants of unknown significance identified in the gene-panel sequencing. We focused on variants with a carrier frequency of ≤2% in the population controls.

As pathogenic variants, we selected all putative LoF (pLoF) variants, defined as stop-gain, frameshift, and essential splice site variants. Additionally, we selected missense and in-frame indel variants that were interpreted as pathogenic or likely-pathogenic in ClinVar [30]. We evaluated the evidence available for these variants in ClinVar and included the variants that were likely to cause a moderately elevated cancer risk.

We examined the missense and in-frame indel variants of unknown significance in search of other potentially pathogenic variants. We tested the association between the variants and cancer risk with Fisher’s exact test using the R environment for statistical computing (version 4.0.3) [31] and two-sided *p* values. We excluded the variants with benign or likely-benign interpretations (including conflicting interpretations) in ClinVar. We selected the variants that were predicted to be deleterious either by Helix [32] or by CADD [33] (phred ≥ 25) and further annotated them with protein domain information from UniProt [34].

### 2.5. Single-Nucleotide Variants and Short Indels in BRCA1/2 Genes

The previous estimate of the *BRCA1/2* carrier frequency in the unselected BC patients was derived from recurrent founder variants [24]. To examine all pathogenic variants found in the unselected BC patients, we selected the pLoF, missense, and in-frame indel variants from the gene-panel sequencing data of the 1356 unselected patients in this study. Of these, we selected the variants that were interpreted as pathogenic or likely-pathogenic in ClinVar as well as previously unreported, likely-pathogenic pLoF variants.

### 2.6. Copy Number Variant Analysis

The CNV data were collected as a part of CNV analysis by BCAC [19], which used the Illumina iCOGS genotyping array with 211,155 probes and OncoArray with 533,631 probes [11,12]. The CNV calling was carried out using CamCNV pipeline as described in detail by Dennis et al. [19,35]. The authors included CNV segments covered by 3 to 200 probes.

We used Ensembl data (release 104) [36] through BioMart [37] and Bedtools (version 2.30.0) [38] to connect the CNV segments to genes and transcripts. As we did not confirm the exact cut-off points of the CNVs, we refer to the CNVs on exonic level in this study. We treated the CNVs leading to the same exonic change as one CNV. The analysis included data from 1137 of the unselected and 612 of the familial patients, with an overlap of 238 patients between the groups, as well as 1025 of the controls.

### 2.7. Multiplex Ligation-Dependent Probe Amplification

All CNVs were validated with the multiplex ligation-dependent probe amplification (MLPA) technique [39]. Details on the used MLPA assays (MRC Holland, Amsterdam, The Netherlands) are given in the Supplementary Table S1. The results were analyzed with the Coffalyser.Net software, version 140721.1958 (MRC Holland).

## 3. Results

### 3.1. Pathogenic Variants in Moderate-Risk Genes

We identified pathogenic or likely-pathogenic variants including CNVs in 112/1356 (8.3%) unselected BC patients, in 90/699 (12.9%) familial BC patients, and in 42/1112 (3.8%) population controls. All of these variants are presented in Table 1. We observed recurrent variants, defined here as variants that have been found in more than one Finnish BC patient, in 101/112 (90.2%) of all of the variant carriers in the unselected series and in 87/90 (96.7%) of the carriers in the familial series. Combining the patient groups, recurrent variants were detected in 163/177 (92.1%) variant carriers. In *PALB2*, *CHEK2*, and *FANCM*, most of the pathogenic burden was covered by a single variant: *PALB2* c.1592delT p.(Leu531CysfsTer30) accounted for 24/27 (88.9%), *CHEK2* c.1100delC p.(Thr367MetfsTer15) for 68/82 (82.9%), and *FANCM* c.5101C>T p.(Gln1701Ter) for 50/58 (86.2%) of the pathogenic variation detected in each gene among the patients.

Besides the major founder variant c.1592delT, we identified four rare pLoF variants in *PALB2*. Three variants were each carried by a single patient and one variant was detected in a population control. In *CHEK2*, the previously reported recurrent variants c.1100delC, c.319+2T>A, and c.444+1G>A covered 80/82 (97.6%) of the pathogenic variation identified in the patients, including one patient who was heterozygous for both c.1100delC and c.319+2T>A. Here, we found three other pathogenic or likely-pathogenic *CHEK2* variants. A deletion of exons three and four and a functionally-damaging missense variant c.433C>T p.(Arg145Trp) [40,41,42] were each detected in a single patient. Additionally, another *CHEK2* pLoF variant was observed in two population controls.

Similarly to *CHEK2*, the previously-known recurrent *FANCM* variants c.5101C>T, c.5791C>T p.(Arg1931Ter), and c.4025_4026del p.(Ser1342Ter) covered 56/58 (96.6%) of the pathogenic variation in this gene among the patients. In addition, we detected one other recurrent *FANCM* variant, c.1491dup p.(Gln498ThrfsTer7), in two patients. In contrast, we observed no major variants in the *ATM* gene. The variants that were detected in more than one individual were the previously-reported c.6908dup p.(Glu2304GlyfsTer69) and a functionally defective missense c.7570G>C p.(Ala2524Pro) [43,44], covering 4/9 (44.4%) of the pathogenic variation. Five other *ATM* variants were found in a single patient each.

In *RAD51C*, we detected the previously-known CNV duplication, which covered the first seven exons of the gene, in three patients and one novel variant in a single patient. To our knowledge, no pathogenic *BARD1* variant has previously been identified in Finnish BC patients; here, we found two *BARD1* pLoF variants, each in a single patient. Additionally, we observed two variants in the OC risk gene *BRIP1* in one patient and one control each. No pathogenic variants were identified in *RAD51D* among the individuals included in this study.

Eight patients carried two or more pathogenic variants in the moderate-risk genes (Supplementary Table S2). With an overlap of three patients between the series, 5/1356 (0.4%) unselected and 6/699 (0.9%) familial BC patients had more than one pathogenic variant. Two of the patients were homozygous for *CHEK2* c.1100delC.

### 3.2. Missense Variants of Uncertain Significance

We tested the missense and in-frame indel variants for BC association and evaluated them based on pathogenicity interpretations submitted to ClinVar and prediction tools. We detected a nominally-significant statistical association for *ATM* c.146C>G p.(Ser49Cys), found in 17/1769 (1.0%) patients compared with 3/1112 (0.3%) controls (OR = 3.59 [95% confidence interval 1.03–19.14], *p* = 0.036); however, this variant is interpreted as benign in ClinVar. Thirty-three missense variants, identified either in patients or controls, passed the selection criteria for potentially pathogenic variants (Supplementary Table S3). All these variants were rare in our BC series and none of them were significantly associated with BC risk (*p* < 0.05).

### 3.3. Pathogenic BRCA1 and BRCA2 Variants among Unselected BC Patients

We found that 24/1356 (1.8%) unselected BC patients had a pathogenic variant in the *BRCA1/2* genes in comparison with 1/1112 (0.09%) population controls (Supplementary Table S4). In more detail, 8/1356 (0.6%) patients had a *BRCA1* and 16/1356 (1.2%) had a *BRCA2* variant. Six of the *BRCA1* and four of the *BRCA2* variants have previously been detected in more than one Finnish BC family (Supplementary Table S4) [23,24,25,45,46,47,48]. Here, these recurrent variants covered 6/8 (75.0%) of the pathogenic variation in *BRCA1* and 11/16 (68.8%) in *BRCA2* among the patients.

### 3.4. Copy Number Variants

We detected preliminary CNVs in the *BRCA1*, *BRCA2*, *ATM*, *CHEK2*, and *RAD51C* genes (Supplementary Table S1). After validation with MLPA, we identified three different pathogenic or likely-pathogenic CNVs: *BRCA1* exon 13 duplication (legacy name exon), *CHEK2* exons 3–4 deletion, and *RAD51C* exons 1–7 duplication were found in 3/1137 (0.3%) patients in the unselected series (Table 1, Supplementary Table S4). In addition, two patients (2/612, 0.3%) in the familial series had the *RAD51C* exons 1–7 duplication (Table 1). *BRCA1* exon 13 and *RAD51C* exons 1–7 duplications have previously been reported in Finnish BC patients [47,48], while the *CHEK2* deletion has not, to our knowledge. Out of all the carriers of any pathogenic or likely-pathogenic variant, 3/134 (2.2%) had a CNV in the unselected patient group and 2/90 (2.2%) in the familial patient group.

Additionally, we found a duplication of exons 62–63 in *ATM* with a frequency of 12/1511 (0.8%) in patients and 9/1025 (0.9%) in controls; hence, it was likely benign. In *BRCA1*, one population control had a large duplication that covered exons 1–20 (legacy name exons) as well as a large section upstream of the gene. A third CNV in *BRCA1* and two CNVs detected in *BRCA2* could not be validated with MLPA and were excluded.

### 3.5. Pathogenic Variant Frequencies in Different Diagnosis Age Groups

We evaluated the frequencies of the pathogenic variants in the unselected series in patients diagnosed with BC at different ages. We observed the variants in the moderate-risk genes in 37/362 (10.2%) patients diagnosed at <50 years of age and in 75/994 (7.5%) patients diagnosed at ≥50 years of age (Supplementary Table S5A). Similarly, 42/536 (7.8%) patients diagnosed at the age of ≥ 60 years carried a pathogenic variant. Excluding the *FANCM* and *BRIP1* variants, 24/362 (6.6%) patients diagnosed at <50 years, 45/994 (4.5%) at ≥50 years, and 24/536 (4.5%) at ≥60 years of age had a pathogenic variant in a moderate-risk gene (Supplementary Table S5B). Additionally, 14/362 (3.9%) patients diagnosed with BC at <50 years and 10/994 (1.0%) at ≥50 years of age had a pathogenic *BRCA1/2* variant.

## 4. Discussion

We have estimated the prevalence of all pathogenic and likely-pathogenic variants in high- and moderate-risk BC and OC susceptibility genes in Finnish BC patients and controls from the Helsinki region. We observed variants in the *PALB2*, *CHEK2*, *ATM*, *BARD1*, *RAD51C*, *BRIP1*, and *FANCM* genes in 8.3% of the unselected BC patients and in 12.9% of the familial BC patients. Excluding the variants found in the putative moderate-risk gene *FANCM* and the OC risk gene *BRIP1*, the carrier frequency was 5.1% in the unselected BC patients and 10.2% in the familial BC patients. In the *BRCA1/2* genes, we identified pathogenic or likely-pathogenic variants in 1.8% of the unselected BC patients.

The overall carrier frequency of pathogenic variants in the validated BC risk genes, observed among the unselected patients, was 6.7%, which is comparable to the results found by other reports. In the BCAC gene-panel sequencing study reported by Dorling et al., about 6.8% of European BC patients had a protein-truncating variant in a BC risk-associated gene, including the *BRCA1/2* and *TP53* genes [3]. Another large population-based study from the United States reported the frequencies of pathogenic variants identified in BC patients with different ethnicities [5]. In that study, approximately 5.0% of the patients carried a variant in a risk gene. It is also worth noticing that we detected pathogenic variants in the validated BC risk genes in 5.3% and 5.0% of the patients diagnosed with BC at 50 and 60 years of age and over, respectively, which might be missed by strict age-based genetic testing.

While the pathogenic variant spectrum in mixed populations is usually wide, our study highlights the strong founder effects in the moderate-risk genes in the studied Finnish BC patients. *PALB2* c.1592delT, *CHEK2* c.1100delC, and *FANCM* c.5101C>T accounted for 88.9%, 82.9%, and 86.2%, respectively, of the pathogenic variation in each gene. Furthermore, the three most common variants in the established risk genes, *PALB2* c.1592delT, *CHEK2* c.1100delC, and *CHEK2* c.319+2T>A, were carried by a notable portion of all patients: 4.1% of the unselected patients and 9.0% of the familial patients. Due to the major recurrent variants, the total frequency of all pathogenic variants in the moderate-risk genes was very similar to the previous estimates analyzing just twelve recurrent variants [25]. We detected new pathogenic variants that were, to our knowledge, previously unreported in the Finnish BC patients, in 1.0% of the unselected patients and in 0.4% of the familial patients. All of these variants were rare and only found in one or two patients each.

Not all previously-known recurrent moderate-risk variants were detected in the current study. We have observed two other recurrent *RAD51C* variants, c.93delG and c.837+1G>A, each in 0.1–0.2% of familial BC patients [25,49]. Additionally, *RAD51D* c.576+1G>A has been found in 0.1% of unselected BC patients and in 0.3% of familial BC patients [25,50], whereas, in this study, no pathogenic *RAD51D* variants were identified. These variants were either not detected by the genotyping and variant calling pipeline or were previously identified due to a larger patient series.

The *BRCA1/2* variant frequency was low among the unselected BC patients, with 0.6% and 1.2% carrying a pathogenic or likely-pathogenic *BRCA1* and *BRCA2* variant, respectively. We have previously identified pathogenic *BRCA1* variants in 1.9% and *BRCA2* variants in 1.1% of 370 additional unselected BC patients who were not included here in the gene-panel sequencing (Supplementary Table S4) [24,25]. For these groups combined, a total of 0.9% and 1.2% of the patients had a *BRCA1* and *BRCA2* variant, respectively. The frequencies are in line with other (population-based) studies [3,5]. Unlike in the moderate-risk genes, the pathogenic variant spectrum detected in the high-penetrance *BRCA1/2* genes in the Finnish BC families is wide with multiple unique variants [25,48]. Nevertheless, strong founder variants have been identified in the *BRCA1/2* genes, especially prominent in *BRCA2* [23,25,48,51,52]. Ten recurrent *BRCA1/2* variants were detected in the unselected patients in the current study. Haplotype analyses have indicated common ancestors for most of these variants in Finland, with two distinct haplotypes detected in the *BRCA2* c.771_775del (previously known as 999del5) carrier families [51,52].

The prevalence of pathogenic CNVs in the BC risk genes has not been explored as extensively as SNPs and short indels; in this study, we investigated the CNVs alongside the other variants. We discovered three likely pathogenic variants, *BRCA1* duplication of exon 13, *CHEK2* deletion of exons 3–4, and *RAD51C* duplication of exons 1–7, which altogether were found in 0.3% of both unselected and familial patients. In comparison, Dennis et al. reported CNV deletions in the BC risk genes in 0.5% of a large series of over 86,000 BC patients [19]. However, these frequencies are likely underestimates, as conclusive CNV calling from array data requires higher probe density than that offered by OncoArray and iCOGS [19]. Hence, the pathogenic CNV spectrum and frequency estimates warrant further studies, also, in Finnish patients. The current CNV detection methods are expensive and time-consuming, and CNVs are often not included in gene panels in clinical testing nor in research. The ongoing development of algorithms and tools to call CNVs from the next-generation sequencing data provides the possibility of the routine inclusion of CNVs in gene-panel testing for comprehensive analysis.

Our results suggest that most carriers among the studied Finnish BC patients could be detected by genotyping the recurrent variants. Of the carriers of a *BRCA1/2* or a moderate-risk variant, 70.8% and 92.1%, respectively, had a recurrent variant in the present study. While gene-panel sequencing is utilized in clinical testing, our results could be used in the designing of population screening of the BC risk variants in Finland. Combined with common low-risk variants into a PRS, the carriers of moderate-risk variants could be provided with improved personalized risk estimates. Recent studies have indicated that the moderate-risk variant carriers with a high PRS may have a BC risk comparable to the carriers of a high-risk variant, whereas, with a low PRS, the carriers may have their risk reduced to the level of the general population [13,14,15]. These estimates could guide cancer prevention strategies for the risk-variant carriers.

## 5. Conclusions

We have estimated the overall prevalence of pathogenic variants in the high- and moderate-risk genes in Finnish BC patients, as well as the contribution of recurrent variants to the pathogenic burden detected in these genes. The combined frequency of the variants was similar to other populations; however, our study highlights the importance of the major recurrent variants in Finnish BC patients, with most of the pathogenic variation resulting from a few variants. Our results are descriptive of the Finnish population and could be utilized in the designing of population screening of the BC risk variants.

## Figures and Tables

**Table 1:** Frequencies of the pathogenic variants in the moderate-risk genes.

	Carrier% per Gene ^3^	All BC ^4^		Familial BC		Unselected BC		Controls	
Variant ^1,2^		Carriers/Total %		Carriers/Total %		Carriers/Total %		Carriers/Total %	
*ATM* c.2554C>T p.(Gln852Ter)	11.11	1/1769	0.06	0/699	0	1/1356	0.07	0/1112	0
*ATM* c.6559G>T p.(Glu2187Ter)	11.11	1/1769	0.06	1/699	0.14	0/1356	0	0/1111	0
***ATM*** **c.6908dup p.(Glu2304GlyfsTer69)**	**11.11**	**1/1764**	**0.06**	**1/699**	**0.14**	**0/1351**	**0**	**2/1109**	**0.18**
***ATM* c.7570G>C p.(Ala2524Pro)**	**33.33**	**3/1768**	**0.17**	**2/699**	**0.29**	**1/1355**	**0.07**	**0/1112**	**0**
*ATM* c.7630-2A>C	11.11	1/1769	0.06	0/699	0	1/1356	0.07	0/1112	0
*ATM* c.8671+2T>A	11.11	1/1766	0.06	0/699	0	1/1353	0.07	0/1107	0
*ATM* c.9139C>T p.(Arg3047Ter)	11.11	1/1769	0.06	0/699	0	1/1356	0.07	0/1112	0
Any *ATM*		9/1769	0.51	4/699	0.57	5/1356	0.37	2/1112	0.18
*BARD1* c.1172C>A p.(Ser391Ter)	50.00	1/1769	0.06	0/699	0	1/1356	0.07	0/1112	0
*BARD1* c.2300_2301del p.(Val767AspfsTer4)	50.00	1/1769	0.06	0/699	0	1/1356	0.07	0/1112	0
Any *BARD1*		2/1769	0.11	0/699	0	2/1356	0.15	0/1112	0
*BRIP1* c.2990_2993del p.(Thr997ArgfsTer61)	100.00	1/1769	0.06	0/699	0	1/1356	0.07	0/1111	0
*BRIP1* c.3219del p.(Ile1074PhefsTer4)	0	0/1769	0	0/699	0	0/1356	0	1/1112	0.09
Any *BRIP1*		1/1769	0.06	0/699	0	1/1356	0.07	1/1112	0.09
***CHEK2* c.319+2T>A**	**13.41**	**11/1769**	**0.62**	**5/699**	**0.72**	**9/1356**	**0.66**	**1/1112**	**0.09**
*CHEK2* ex3-4del	1.22	1/1511	0.07	0/612	0	1/1137	0.09	0/1025	0
*CHEK2* c.433C>T p.(Arg145Trp)	1.22	1/1769	0.06	0/699	0	1/1356	0.07	0/1112	0
***CHEK2* c.444+1G>A**	**1.22**	**1/1769**	**0.06**	**0/699**	**0**	**1/1356**	**0.07**	**0/1112**	**0**
***CHEK2*** **c.1100del p.(Thr367MetfsTer15)**	**82.93**	**68/1769**	**3.84**	**43/699**	**6.15**	**38/1356**	**2.80**	**14/1112**	**1.26**
*CHEK2* c.1368dup p.(Glu457ArgfsTer33)	0	0/1768	0	0/699	0	0/1355	0	2/1112	0.18
Any *CHEK2*		81/1769	4.58	47/699	6.72	49/1356	3.61	17/1112	1.53
***FANCM* c.1491dup p.(Gln498ThrfsTer7)**	**3.45**	**2/1769**	**0.11**	**0/699**	**0**	**2/1356**	**0.15**	**1/1112**	**0.09**
***FANCM*** **c.4025_4026del p.(Ser1342Ter)**	**1.72**	**1/1769**	**0.06**	**1/699**	**0.14**	**0/1356**	**0**	**1/1112**	**0.09**
***FANCM*** **c.5101C>T p.(Gln1701Ter)**	**86.21**	**50/1768**	**2.83**	**19/699**	**2.72**	**40/1355**	**2.95**	**15/1107**	**1.36**
***FANCM*** **c.5791C>T p.(Arg1931Ter)**	**8.62**	**5/1755**	**0.28**	**2/696**	**0.29**	**3/1345**	**0.22**	**3/1092**	**0.27**
Any *FANCM*		58/1769	3.28	22/699	3.15	45/1356	3.32	20/1112	1.80
*PALB2* c.172_175del p.(Gln60ArgfsTer7)	0	0/1768	0	0/698	0	0/1356	0	1/1112	0.09
*PALB2* c.1056_1057del p.(Lys353IlefsTer7)	3.70	1/1769	0.06	1/699	0.14	0/1356	0	0/1112	0
*PALB2* c.1387del p.(Ile463LeufsTer22)	3.70	1/1762	0.06	0/698	0	1/1350	0.07	0/1097	0
***PALB2*** **c.1592del p.(Leu531CysfsTer30)**	**88.89**	**24/1768**	**1.36**	**16/699**	**2.29**	**10/1355**	**0.74**	**1/1109**	**0.09**
*PALB2* c.2719G>T p.(Glu907Ter)	3.70	1/1769	0.06	0/699	0	1/1356	0.07	0/1112	0
Any *PALB2*		27/1769	1.53	17/699	2.43	12/1356	0.88	2/1112	0.18
***RAD51C* ex1-7dup**	**75.00**	**3/1511**	**0.20**	**2/612**	**0.33**	**1/1137**	**0.09**	**0/1025**	**0**
*RAD51C* c.338dup p.(Gly114TrpfsTer41)	25.00	1/1766	0.06	1/698	0.14	0/1354	0	0/1109	0
Any *RAD51C*		4/1769	0.23	3/699	0.43	1/1356	0.07	0/1112	0
Total ^5^		177/1769	10.01	90/699	12.88	112/1356	8.26	42/1112	3.78

^1^ Reference transcripts: *ATM* NM_000051.3, *BARD1* NM_000465.2, *BRIP1* NM_032043.2, *CHEK2* NM_007194.3, *FANCM* NM_020937.2, *PALB2* NM_024675.3, and *RAD51C* NM_058216.2. ^2^ Variants that recur in Finnish BC patients are in bold. ^3^ In all BC patients. ^4^ Total number of patients after removing the overlap of 286 patients between the familial and the unselected BC series. ^5^ Individuals with two or more pathogenic variants were counted once in the total frequencies.

## Data Availability

The data presented in this study are available on request from the corresponding author. The data are not publicly available due to privacy or ethical restrictions. Access to the BCAC data can be applied by contacting the BCAC Coordinator (https://bcac.ccge.medschl.cam.ac.uk).

## Supplementary Materials

The following supporting information can be downloaded at: https://www.mdpi.com/article/10.3390/cancers14246158/s1, Table S1: Detected and validated copy number variants; Table S2: Carriers of more than one pathogenic variant; Table S3: Missenses of uncertain significance; Table S4: Pathogenic variants in the *BRCA1* and *BRCA2* genes; Table S5: Frequencies of pathogenic variants identified in patients diagnosed with breast cancer at different ages; Supplementary References.

## References

[1] Pitkäniemi J., Malila N., Tanskanen T., Degerlund H., Heikkinen S., Seppä K. (2022). Cancer in Finland 2020.

[2] Lee K., Seifert B.A., Shimelis H., Ghosh R., Crowley S.B., Carter N.J., Doonanco K., Foreman A.K., Ritter D.I., Jimenez S. (2019). Clinical validity assessment of genes frequently tested on hereditary breast and ovarian cancer susceptibility sequencing panels. Genet. Med..

[3] Dorling L., Carvalho S., Allen J., Gonzalez-Neira A., Luccarini C., Wahlstrom C., Pooley K.A., Parsons M.T., Fortuno C., Breast Cancer Association Consortium (2021). Breast Cancer Risk Genes-Association Analysis in More than 113,000 Women. N. Engl. J. Med..

[4] Kuchenbaecker K.B., Hopper J.L., Barnes D.R., Phillips K.A., Mooij T.M., Roos-Blom M.J., Jervis S., van Leeuwen F.E., Milne R.L., Andrieu N. (2017). Risks of Breast, Ovarian, and Contralateral Breast Cancer for BRCA1 and BRCA2 Mutation Carriers. JAMA.

[5] Hu C., Hart S.N., Gnanaolivu R., Huang H., Lee K.Y., Na J., Gao C., Lilyquist J., Yadav S., Boddicker N.J. (2021). A Population-Based Study of Genes Previously Implicated in Breast Cancer. N. Engl. J. Med..

[6] Lilyquist J., LaDuca H., Polley E., Davis B.T., Shimelis H., Hu C., Hart S.N., Dolinsky J.S., Couch F.J., Goldgar D.E. (2017). Frequency of mutations in a large series of clinically ascertained ovarian cancer cases tested on multi-gene panels compared to reference controls. Gynecol. Oncol..

[7] Suszynska M., Ratajska M., Kozlowski P. (2020). BRIP1, RAD51C, and RAD51D mutations are associated with high susceptibility to ovarian cancer: Mutation prevalence and precise risk estimates based on a pooled analysis of ~30,000 cases. J. Ovarian Res..

[8] Shimelis H., LaDuca H., Hu C., Hart S.N., Na J., Thomas A., Akinhanmi M., Moore R.M., Brauch H., Cox A. (2018). Triple-Negative Breast Cancer Risk Genes Identified by Multigene Hereditary Cancer Panel Testing. J. Natl. Cancer Inst..

[9] Kiiski J.I., Pelttari L.M., Khan S., Freysteinsdottir E.S., Reynisdottir I., Hart S.N., Shimelis H., Vilske S., Kallioniemi A., Schleutker J. (2014). Exome sequencing identifies FANCM as a susceptibility gene for triple-negative breast cancer. Proc. Natl. Acad. Sci. USA.

[10] Peterlongo P., Figlioli G., Deans A.J., Couch F.J. (2021). Protein truncating variants in FANCM and risk for ER-negative/triple negative breast cancer. NPJ Breast Cancer.

[11] Michailidou K., Hall P., Gonzalez-Neira A., Ghoussaini M., Dennis J., Milne R.L., Schmidt M.K., Chang-Claude J., Bojesen S.E., Bolla M.K. (2013). Large-scale genotyping identifies 41 new loci associated with breast cancer risk. Nat. Genet..

[12] Michailidou K., Lindstrom S., Dennis J., Beesley J., Hui S., Kar S., Lemacon A., Soucy P., Glubb D., Rostamianfar A. (2017). Association analysis identifies 65 new breast cancer risk loci. Nature.

[13] Gallagher S., Hughes E., Wagner S., Tshiaba P., Rosenthal E., Roa B.B., Kurian A.W., Domchek S.M., Garber J., Lancaster J. (2020). Association of a Polygenic Risk Score with Breast Cancer Among Women Carriers of High- and Moderate-Risk Breast Cancer Genes. JAMA Netw. Open.

[14] Mars N., Widen E., Kerminen S., Meretoja T., Pirinen M., Della Briotta Parolo P., Palta P., FinnGen, Palotie A., Kaprio J. (2020). The role of polygenic risk and susceptibility genes in breast cancer over the course of life. Nat. Commun..

[15] Gao C., Polley E.C., Hart S.N., Huang H., Hu C., Gnanaolivu R., Lilyquist J., Boddicker N.J., Na J., Ambrosone C.B. (2021). Risk of Breast Cancer Among Carriers of Pathogenic Variants in Breast Cancer Predisposition Genes Varies by Polygenic Risk Score. J. Clin. Oncol..

[16] Collins R.L., Brand H., Karczewski K.J., Zhao X., Alfoldi J., Francioli L.C., Khera A.V., Lowther C., Gauthier L.D., Wang H. (2020). A structural variation reference for medical and population genetics. Nature.

[17] The BRCA1 Exon 13 Duplication Screening Group (2000). The exon 13 duplication in the BRCA1 gene is a founder mutation present in geographically diverse populations. Am. J. Hum. Genet..

[18] Cybulski C., Wokolorczyk D., Huzarski T., Byrski T., Gronwald J., Gorski B., Debniak T., Masojc B., Jakubowska A., van de Wetering T. (2007). A deletion in CHEK2 of 5,395 bp predisposes to breast cancer in Poland. Breast Cancer Res. Treat..

[19] Dennis J., Tyrer J.P., Walker L.C., Michailidou K., Dorling L., Bolla M.K., Wang Q., Ahearn T.U., Andrulis I.L., Anton-Culver H. (2022). Rare germline copy number variants (CNVs) and breast cancer risk. Commun. Biol..

[20] Walker L.C., Wiggins G.A., Pearson J.F. (2015). The Role of Constitutional Copy Number Variants in Breast Cancer. Microarrays.

[21] Lim E.T., Wurtz P., Havulinna A.S., Palta P., Tukiainen T., Rehnstrom K., Esko T., Magi R., Inouye M., Lappalainen T. (2014). Distribution and medical impact of loss-of-function variants in the Finnish founder population. PLoS Genet..

[22] Kaariainen H., Muilu J., Perola M., Kristiansson K. (2017). Genetics in an isolated population like Finland: A different basis for genomic medicine?. J. Community Genet..

[23] Vehmanen P., Friedman L.S., Eerola H., McClure M., Ward B., Sarantaus L., Kainu T., Syrjakoski K., Pyrhonen S., Kallioniemi O.P. (1997). Low proportion of BRCA1 and BRCA2 mutations in Finnish breast cancer families: Evidence for additional susceptibility genes. Hum. Mol. Genet..

[24] Syrjakoski K., Vahteristo P., Eerola H., Tamminen A., Kivinummi K., Sarantaus L., Holli K., Blomqvist C., Kallioniemi O.P., Kainu T. (2000). Population-based study of BRCA1 and BRCA2 mutations in 1035 unselected Finnish breast cancer patients. J. Natl. Cancer Inst..

[25] Nurmi A., Muranen T.A., Pelttari L.M., Kiiski J.I., Heikkinen T., Lehto S., Kallioniemi A., Schleutker J., Butzow R., Blomqvist C. (2019). Recurrent moderate-risk mutations in Finnish breast and ovarian cancer patients. Int. J. Cancer.

[26] Kilpivaara O., Bartkova J., Eerola H., Syrjakoski K., Vahteristo P., Lukas J., Blomqvist C., Holli K., Heikkila P., Sauter G. (2005). Correlation of CHEK2 protein expression and c.1100delC mutation status with tumor characteristics among unselected breast cancer patients. Int. J. Cancer.

[27] Fagerholm R., Hofstetter B., Tommiska J., Aaltonen K., Vrtel R., Syrjakoski K., Kallioniemi A., Kilpivaara O., Mannermaa A., Kosma V.M. (2008). NAD(P)H:quinone oxidoreductase 1 NQO1*2 genotype (P187S) is a strong prognostic and predictive factor in breast cancer. Nat. Genet..

[28] Eerola H., Blomqvist C., Pukkala E., Pyrhonen S., Nevanlinna H. (2000). Familial breast cancer in southern Finland: How prevalent are breast cancer families and can we trust the family history reported by patients?. Eur. J. Cancer.

[29] Vahteristo P., Bartkova J., Eerola H., Syrjakoski K., Ojala S., Kilpivaara O., Tamminen A., Kononen J., Aittomaki K., Heikkila P. (2002). A CHEK2 genetic variant contributing to a substantial fraction of familial breast cancer. Am. J. Hum. Genet..

[30] Landrum M.J., Lee J.M., Benson M., Brown G.R., Chao C., Chitipiralla S., Gu B., Hart J., Hoffman D., Jang W. (2018). ClinVar: Improving access to variant interpretations and supporting evidence. Nucleic Acids Res..

[31] R Core Team (2020). R: A Language and Environment for Statistical Computing.

[32] Vroling B., Heijl S. (2021). White paper: The Helix Pathogenicity Prediction Platform. arXiv.

[33] Kircher M., Witten D.M., Jain P., O’Roak B.J., Cooper G.M., Shendure J. (2014). A general framework for estimating the relative pathogenicity of human genetic variants. Nat. Genet..

[34] UniProt C. (2021). The universal protein knowledgebase in 2021. Nucleic Acids Res..

[35] Dennis J., Walker L., Tyrer J., Michailidou K., Easton D.F. (2021). Detecting rare copy number variants from Illumina genotyping arrays with the CamCNV pipeline: Segmentation of z-scores improves detection and reliability. Genet. Epidemiol..

[36] Howe K.L., Achuthan P., Allen J., Allen J., Alvarez-Jarreta J., Amode M.R., Armean I.M., Azov A.G., Bennett R., Bhai J. (2021). Ensembl 2021. Nucleic Acids Res..

[37] Kinsella R.J., Kahari A., Haider S., Zamora J., Proctor G., Spudich G., Almeida-King J., Staines D., Derwent P., Kerhornou A. (2011). Ensembl BioMarts: A hub for data retrieval across taxonomic space. Database.

[38] Quinlan A.R., Hall I.M. (2010). BEDTools: A flexible suite of utilities for comparing genomic features. Bioinformatics.

[39] Schouten J.P., McElgunn C.J., Waaijer R., Zwijnenburg D., Diepvens F., Pals G. (2002). Relative quantification of 40 nucleic acid sequences by multiplex ligation-dependent probe amplification. Nucleic Acids Res..

[40] Lee S.B., Kim S.H., Bell D.W., Wahrer D.C., Schiripo T.A., Jorczak M.M., Sgroi D.C., Garber J.E., Li F.P., Nichols K.E. (2001). Destabilization of CHK2 by a missense mutation associated with Li-Fraumeni Syndrome. Cancer Res..

[41] Wu X., Webster S.R., Chen J. (2001). Characterization of tumor-associated Chk2 mutations. J. Biol. Chem..

[42] Delimitsou A., Fostira F., Kalfakakou D., Apostolou P., Konstantopoulou I., Kroupis C., Papavassiliou A.G., Kleibl Z., Stratikos E., Voutsinas G.E. (2019). Functional characterization of CHEK2 variants in a Saccharomyces cerevisiae system. Hum. Mutat..

[43] Allinen M., Launonen V., Laake K., Jansen L., Huusko P., Kaariainen H., Borresen-Dale A.L., Winqvist R. (2002). ATM mutations in Finnish breast cancer patients. J. Med. Genet..

[44] Pylkas K., Tommiska J., Syrjakoski K., Kere J., Gatei M., Waddell N., Allinen M., Karppinen S.M., Rapakko K., Kaariainen H. (2007). Evaluation of the role of Finnish ataxia-telangiectasia mutations in hereditary predisposition to breast cancer. Carcinogenesis.

[45] Huusko P., Paakkonen K., Launonen V., Poyhonen M., Blanco G., Kauppila A., Puistola U., Kiviniemi H., Kujala M., Leisti J. (1998). Evidence of founder mutations in Finnish BRCA1 and BRCA2 families. Am. J. Hum. Genet..

[46] Hartikainen J.M., Kataja V., Pirskanen M., Arffman A., Ristonmaa U., Vahteristo P., Ryynanen M., Heinonen S., Kosma V.M., Mannermaa A. (2007). Screening for BRCA1 and BRCA2 mutations in Eastern Finnish breast/ovarian cancer families. Clin. Genet..

[47] Pelttari L.M., Shimelis H., Toiminen H., Kvist A., Torngren T., Borg A., Blomqvist C., Butzow R., Couch F., Aittomaki K. (2018). Gene-panel testing of breast and ovarian cancer patients identifies a recurrent RAD51C duplication. Clin. Genet..

[48] Pallonen T.A., Lempiainen S.M.M., Joutsiniemi T.K., Aaltonen R.I., Pohjola P.E., Kankuri-Tammilehto M.K. (2022). Genetic, clinic and histopathologic characterization of BRCA-associated hereditary breast and ovarian cancer in southwestern Finland. Sci. Rep..

[49] Pelttari L.M., Heikkinen T., Thompson D., Kallioniemi A., Schleutker J., Holli K., Blomqvist C., Aittomaki K., Butzow R., Nevanlinna H. (2011). RAD51C is a susceptibility gene for ovarian cancer. Hum. Mol. Genet..

[50] Pelttari L.M., Kiiski J., Nurminen R., Kallioniemi A., Schleutker J., Gylfe A., Aaltonen L.A., Leminen A., Heikkila P., Blomqvist C. (2012). A Finnish founder mutation in RAD51D: Analysis in breast, ovarian, prostate, and colorectal cancer. J. Med. Genet..

[51] Sarantaus L., Huusko P., Eerola H., Launonen V., Vehmanen P., Rapakko K., Gillanders E., Syrjakoski K., Kainu T., Vahteristo P. (2000). Multiple founder effects and geographical clustering of BRCA1 and BRCA2 families in Finland. Eur. J. Hum. Genet..

[52] Barkardottir R.B., Sarantaus L., Arason A., Vehmanen P., Bendahl P.O., Kainu T., Syrjakoski K., Krahe R., Huusko P., Pyrhonen S. (2001). Haplotype analysis in Icelandic and Finnish BRCA2 999del5 breast cancer families. Eur. J. Hum. Genet..

