# Supplementary material for: Pathogenic Variant Spectrum in Breast Cancer Risk Genes in Finnish Patients
Source: Cancers (Basel). 2022 Dec 14;14(24):6158. doi: 10.3390/cancers14246158 (PMC9776204; doi:10.3390/cancers14246158)
Supplement: Supplementary file 1 [file cancers-14-06158-s001.zip › cancers-2052010-supplementary.pdf]

Pathogenic Variant Spectrum in Breast Cancer Risk Genes in Finnish Patients

Anna K. Nurmi, Maija Suvanto, Joe Dennis, Kristiina Aittomäki, Carl Blomqvist and Heli Nevanlinna

Supplementary Information

Table S1. Detected and validated copy number variants

Table S2. Carriers of more than one pathogenic variant

Table S3. Missenses of uncertain significance

Table S4. Pathogenic variants in the *BRCA1* and *BRCA2* genes

Table S5. Frequencies of pathogenic variants identified in patients diagnosed with breast cancer at different ages

Supplementary References (Table S4)

Table S1. Detected and validated copy number variants

	CamCNV pipeline called regions (human genome build 19)									Multiplex ligation-dependent probe amplification				
Gene	Chr	Start	End	Length	CNV call	Array	Number of probes	Patients	Controls	Validated	Exons affected	Assay	Patients	Controls
BRCA1	17	41209139	41585657	376519	Dup	iCOGS	106	0	1	Yes	1-20 [1]	P002	0	1
BRCA1	17	41218805	41223094	4290	Dup	iCOGS	10	3	0	No	NA	P002	0	0
BRCA1	17	41219619	41223094	3476	Dup	iCOGS	8	1	0	No	NA	P002	0	0
BRCA1	17	41232293	41234592	2300	Dup	OncoArray	6	1	0	Yes	13 [1]	P002	1	0
BRCA2	13	32911796	32912402	607	Dup	iCOGS	7	1	0	No	NA	P090	0	0
BRCA2	13	32912073	32912402	330	Del	iCOGS	5	0	1	No	NA	P090	0	0
ATM	11	108234836	108240069	5234	Dup	iCOGS	5	0	1	Yes	62-63	P042	0	1
ATM	11	108234836	108244114	9279	Dup	iCOGS	6	11	5	Yes	62-63	P041, P042	11	5
ATM	11	108234836	108261261	26426	Dup	iCOGS	8	0	1	Yes	62-63	P042	0	1
ATM	11	108235879	108243476	7598	Dup	OncoArray	7	0	2	Yes	62-63	P042	0	2
ATM	11	108237662	108244114	6453	Dup	iCOGS	4	1	0	Yes	62-63	P041, P042	1	0
CHEK2	22	29121019	29121326	308	Del	iCOGS	4	1	0	Yes	3-4	P190	1	0
RAD51C	17	56757584	56802141	44558	Dup	iCOGS	25	2	0	Yes	1-7	P260	2	0
RAD51C	17	56757584	56808956	51373	Dup	iCOGS	27	1	0	Yes	1-7	P260	1	0

[1] *BRCA1* legacy name exon.

Table S2. Carriers of more than one pathogenic variant

Patient	Variants	Patient series
1	*PALB2* c.1592del, *FANCM* c.5101C>T	Familial BC
2	*CHEK2* c.1100del, *FANCM* c.5101C>T	Familial BC
3	*CHEK2* c.1100del homozygous	Familial BC
4	*CHEK2* c.1100del homozygous	Familial BC, unselected BC
5	*CHEK2* c.1100del, *CHEK2* c.319+2T>A	Familial BC, unselected BC
6	*CHEK2* c.1100del, *FANCM* c.5101C>T	Familial BC, unselected BC
7	*BRCA2* c.7480C>T, *CHEK2* c.1100del, *FANCM* c.5101C>T	Unselected BC
8	*BRCA1* c.3626del, *CHEK2* c.319+2T>A	Unselected BC
9	*PALB2* c.1592del, *FANCM* c.5101C>T	Unselected BC

Table S3. Missenses of uncertain significance

Gene	Variant [1]	Domain or region	All BC patients [2] Carriers/total (%)	Familial BC patients Carriers/total (%)	Unselected BC patients Carriers/total (%)	Controls Carriers/total (%)	Pathogenicity interpretation ClinVar	Predictions Helix	CADD
ATM	c.1727T>C, p.(Ile576Thr)	NA	1/1769 (0.06)	1/699 (0.14)	1/1356 (0.07)	2/1112 (0.18)	Uncertain significance	Benign, medium confidence	25
ATM	c.1832T>A, p.(Ile611Asn)	NA	1/1769 (0.06)	1/699 (0.14)	0/1356 (0)	1/1112 (0.09)	NA	Benign, medium confidence	25.4
ATM	c.3247C>T, p.(His1083Tyr)	NA	0/1732 (0)	0/693 (0)	0/1324 (0)	1/1062 (0.09)	Uncertain significance	Benign, medium confidence	25.9
ATM	c.5296C>T, p.(Pro1766Ser)	NA	3/1769 (0.17)	1/699 (0.14)	2/1356 (0.15)	0/1112 (0)	NA	Benign, medium confidence	25
ATM	c.5551C>T, p.(Leu1851Phe)	NA	1/1769 (0.06)	0/699 (0)	1/1356 (0.07)	0/1112 (0)	Uncertain significance	Benign, high confidence	25.9
ATM	c.6313A>G, p.(Arg2105Gly)	FAT	4/1706 (0.23)	1/679 (0.15)	4/1307 (0.31)	6/1042 (0.58)	Uncertain significance	Benign, medium confidence	31
ATM	c.7375C>G, p.(Arg2459Gly)	FAT	1/1769 (0.06)	0/699 (0)	1/1356 (0.07)	0/1111 (0)	Uncertain significance	Benign, low confidence	26.6
ATM	c.7547T>G, p.(Phe2516Cys)	FAT	1/1768 (0.06)	0/699 (0)	1/1355 (0.07)	2/1112 (0.18)	Uncertain significance	Deleterious, low confidence	26.3
ATM	c.8734A>G, p.(Arg2912Gly)	PI3K/PI4K	1/1769 (0.06)	0/699 (0)	1/1356 (0.07)	0/1112 (0)	Uncertain significance	Deleterious, low confidence	28
ATM	c.9094G>C, p.(Val3032Leu)	FATC	2/1769 (0.11)	1/699 (0.14)	2/1356 (0.15)	1/1112 (0.09)	Uncertain significance	Deleterious, low confidence	24.7
BARD1	c.1307C>G, p.(Ser436Cys)	ANK 1 repeat	1/1769 (0.06)	0/699 (0)	1/1356 (0.07)	0/1112 (0)	Uncertain significance	Benign, low confidence	26.6
BARD1	c.1829C>A, p.(Pro610His)	BRCT 1	1/1749 (0.06)	0/692 (0)	1/1342 (0.07)	0/1075 (0)	NA	Benign, low confidence	26.1
BRIP1	c.257G>A, p.(Cys86Tyr)	Helicase ATP-binding	0/1768 (0)	0/699 (0)	0/1355 (0)	1/1107 (0.09)	Uncertain significance	Deleterious, low confidence	24.9
BRIP1	c.806C>T, p.(Ser269Leu)	Helicase ATP-binding	1/1769 (0.06)	0/699 (0)	1/1356 (0.07)	0/1112 (0)	Uncertain significance	Benign, low confidence	28.6
BRIP1	c.1061C>T, p.(Thr354Ile)	Helicase ATP-binding	1/1769 (0.06)	1/699 (0.14)	0/1356 (0)	0/1112 (0)	Uncertain significance	Benign, medium confidence	26.5
BRIP1	c.1774T>C, p.(Trp592Arg)	NA	1/1769 (0.06)	0/699 (0)	1/1356 (0.07)	0/1112 (0)	NA	Deleterious, high confidence	28.1
BRIP1	c.2275G>A, p.(Ala759Thr)	NA	0/1769 (0)	0/699 (0)	0/1356 (0)	1/1112 (0.09)	NA	Deleterious, low confidence	29.1
CHEK2	c.607G>T, p.(Asp203Tyr)	NA	0/1737 (0)	0/693 (0)	0/1326 (0)	2/1061 (0.19)	NA	Benign, low confidence	32
CHEK2	c.715G>A, p.(Glu239Lys)	Protein kinase	1/1743 (0.06)	0/694 (0)	1/1334 (0.07)	0/1062 (0)	Uncertain significance	Deleterious, low confidence	23.3
CHEK2	c.1022A>C, p.(Asn341Thr)	Protein kinase	1/1769 (0.06)	0/699 (0)	1/1356 (0.07)	0/1112 (0)	Uncertain significance	Deleterious, low confidence	26
CHEK2	c.1427C>T, p.(Thr476Met)	Protein kinase	2/1761 (0.11)	1/698 (0.14)	1/1349 (0.07)	0/1108 (0)	Likely pathogenic, Uncertain significance	Deleterious, low confidence	26.3
CHEK2	c.1447C>T, p.(His483Tyr)	Protein kinase	1/1761 (0.06)	1/698 (0.14)	1/1349 (0.07)	0/1107 (0)	Uncertain significance	Deleterious, low confidence	28.2

Table S3. continues

Gene	Variant [1]	Domain or region	All BC patients [2] Carriers/total (%)	Familial BC patients Carriers/total (%)	Unselected BC patients Carriers/total (%)	Controls Carriers/total (%)	Pathogenicity interpretation ClinVar	Predictions Helix	CADD
FANCM	c.163G>A, p.(Asp55Asn)	NA	4/1769 (0.23)	2/699 (0.29)	3/1356 (0.22)	0/1112 (0)	Uncertain significance	Benign, high confidence	27.6
FANCM	c.269C>T, p.(Pro90Leu)	NA	1/1769 (0.06)	0/699 (0)	1/1356 (0.07)	0/1112 (0)	Uncertain significance	Benign, high confidence	26.8
FANCM	c.1508T>A, p.(Ile503Asn)	Helicase C-terminal	1/1769 (0.06)	0/699 (0)	1/1356 (0.07)	0/1112 (0)	NA	Benign, low confidence	27
FANCM	c.1597C>T, p.(Arg533Cys)	Helicase C-terminal	0/1769 (0)	0/699 (0)	0/1356 (0)	1/1112 (0.09)	Uncertain significance	Benign, low confidence	32
PALB2	c.101G>A, p.(Arg34His)	DNA-binding	1/1769 (0.06)	0/699 (0)	1/1356 (0.07)	0/1112 (0)	Uncertain significance	Benign, high confidence	28.2
PALB2	c.2689C>T, p.(Leu897Phe)	WD 1 repeat	1/1769 (0.06)	1/699 (0.14)	0/1356 (0)	0/1112 (0)	Uncertain significance	Benign, low confidence	26.3
PALB2	c.3520G>A, p.(Gly1174Arg)	WD 7 repeat	0/1769 (0)	0/699 (0)	0/1356 (0)	1/1112 (0.09)	Uncertain significance	Deleterious, low confidence	29.6
RAD51C	c.772C>T, p.(Arg258Cys)	NA	0/1769 (0)	0/699 (0)	0/1356 (0)	1/1112 (0.09)	Uncertain significance	Deleterious, low confidence	29.7
RAD51C	c.1103G>A, p.(Arg368Gln)	Nuclear localization signal	1/1758 (0.06)	0/695 (0)	1/1349 (0.07)	0/1091 (0)	Uncertain significance	Benign, low confidence	25.6
RAD51D	c.287G>T, p.(Gly96Val)	NA	1/1769 (0.06)	0/699 (0)	1/1356 (0.07)	2/1112 (0.18)	NA	Deleterious, low confidence	25.4
RAD51D	c.899G>A, p.(Arg300Gln)	NA	1/1769 (0.06)	1/699 (0.14)	0/1356 (0)	0/1111 (0)	Uncertain significance	Deleterious, low confidence	28.1

[1] Reference transcripts: *ATM* NM_000051.3, *BARD1* NM_000465.2, *BRIP1* NM_032043.2, *CHEK2* NM_007194.3, *FANCM* NM_020937.2, *PALB2* NM_024675.3, *RAD51C* NM_058216.2, and *RAD51D* NM_002878.3.

[2] All breast cancer patients after removing the overlap of 286 individuals between the familial and the unselected patient groups.

Table S4. Pathogenic variants in the *BRCA1* and *BRCA2* genes

Gene	Variant [1]	ClinVar	Combined Unselected BC patients Carriers/total	%	Gene-panel sequencing Unselected BC patients Carriers/total	%	Previous studies Unselected BC patients Carriers/total	%	Gene-panel sequencing Controls Carriers/total	%	Supplementary References
BRCA1	c.485_486del, p.(Val162GlufsTer19)	Pathogenic [3]	1/1726	0.06	1/1351	0.07	–		0/1111	0	–
BRCA1	c.594_597del, p.(Ser198ArgfsTer35)	Pathogenic/Likely pathogenic	1/1726	0.06	–		1/370	0.27	–		[1]
BRCA1	c.667_668del, p.(Lys223GlyfsTer4)	Pathogenic	1/1726	0.06	–		1/370	0.27	–		[1]
BRCA1	c.3485del, p.(Asp1162ValfsTer48) [2]	Pathogenic [3]	2/1726	0.12	1/1356	0.07	1/370	0.27	0/1112	0	[1-5]
BRCA1	c.3626del, p.(Leu1209Ter) [2]	Pathogenic [3]	1/1726	0.06	1/1356	0.07	–		0/1112	0	[1,2,4-6]
BRCA1	c.4097-2A>G [2]	Pathogenic [3]	3/1726	0.17	1/1356	0.07	2/370	0.54	0/1112	0	[1-7]
BRCA1	c.4327C>T, p.(Arg1443Ter) [2]	Pathogenic [3]	1/1726	0.06	–		1/370	0.27	–		[1-4]
BRCA1	c.4357+1G>A [2]	Pathogenic [3]	1/1726	0.06	1/1313	0.08	–		0/1052	0	[5]
BRCA1	ex13dup [2]	Pathogenic	1/1726	0.06	1/1137	0.09	–		0/1025	0	[5,8]
BRCA1	c.4480G>T, p.(Glu1494Ter)	Pathogenic [3]	1/1726	0.06	–		1/370	0.27	–		[1]
BRCA1	c.5209dup, p.(Arg1737LysfsTer93)	Pathogenic [3]	1/1726	0.06	1/1356	0.07	–		0/1110	0	[1]
BRCA1	c.5266dup, p.(Gln1756ProfsTer74) [2]	Pathogenic [3]	1/1726	0.06	1/1353	0.07	–		0/1097	0	[1,2,5]
BRCA2	c.376C>T, p.(Gln126Ter)	Pathogenic/Likely pathogenic	1/1726	0.06	1/1355	0.07	–		0/1112	0	–
BRCA2	c.517-2A>G	Pathogenic [3]	1/1726	0.06	1/1356	0.07	–		0/1112	0	[1]
BRCA2	c.771_775del, p.(Asn257LysfsTer17) [2]	Pathogenic [3]	4/1726	0.23	2/1324	0.15	2/370	0.54	0/1060	0	[1-3,5-7,9]
BRCA2	c.1689G>A, p.(Trp563Ter)	Pathogenic [3]	1/1726	0.06	1/1314	0.08	–		0/1068	0	–
BRCA2	c.6275_6276del, p.(Leu2092ProfsTer7) [2]	Pathogenic [3]	1/1726	0.06	1/1352	0.07	–		0/1106	0	[3-7]
BRCA2	c.7480C>T, p.(Arg2494Ter) [2]	Pathogenic [3]	6/1726	0.35	6/1356	0.44	–		1/1112	0.09	[1-5]
BRCA2	c.8177A>G, p.(Tyr2726Cys)	Pathogenic/Likely pathogenic	1/1726	0.06	1/1356	0.07	–		0/1112	0	–
BRCA2	c.9118-2A>G [2]	Pathogenic [3]	4/1726	0.23	2/1356	0.15	2/370	0.54	0/1109	0	[1-6]
BRCA2	c.9692_9699dup, p.(Met3234HisfsTer18)	NA	1/1726	0.06	1/1356	0.07	–		0/1112	0	–
Any *BRCA1*			15/1726	0.87	8/1356	0.59	7/370	1.89	0/1112	0	
Any *BRCA2*			20/1726	1.16	16/1356	1.18	4/370	1.08	1/1112	0.09	
Total			35/1726	2.03	24/1356	1.77	11/370	2.97	1/1112	0.09	

[1] Reference transcripts: *BRCA1* NM_007294.3 and *BRCA2* NM_000059.3.
[2] Variants that recur in Finnish breast cancer patients.
[3] Pathogenicity reviewed by expert panel.

Table S5. Frequencies of pathogenic variants identified in patients diagnosed with breast cancer at different ages

A. Carriers of a *BRCA1/2* or a moderate-risk variant in the unselected patient group

Age of diagnosis	Total	*BRCA1/2* variant	%	Moderate-risk variant	%	Combined [1]	%
≤ 39	78	7	9.0	7	9.0	14	17.9
40-49	284	7	2.5	30	10.6	37	13.0
50-59	458	7	1.5	33	7.2	38	8.3
60-69	307	2	0.7	22	7.2	24	7.8
70-79	161	1	0.6	15	9.3	16	9.9
≥ 80	68	0	0	5	7.4	5	7.4
Total	1356	24	1.8	112	8.3	134	9.9

B. Carriers of a *BRCA1/2* or a moderate-risk variant, excluding the *FANCM* and *BRIP1* variants, in the unselected patient group

Age of diagnosis	Total	*BRCA1/2* variant	%	Moderate-risk variant	%	Combined [1]	%
≤ 39	78	7	9.0	6	7.7	13	16.7
40-49	284	7	2.5	18	6.3	25	8.8
50-59	458	7	1.5	21	4.6	26	5.7
60-69	307	2	0.7	11	3.6	13	4.2
70-79	161	1	0.6	10	6.2	11	6.8
≥ 80	68	0	0.0	3	4.4	3	4.4
Total	1356	24	1.8	69	5.1	91	6.7

[1] The patients with both a *BRCA1/2* and a moderate-risk variant were included once in the combined frequencies.

Supplementary References (Table S4)

1. Nurmi, A.; Muranen, T.A.; Pelttari, L.M.; Kiiski, J.I.; Heikkinen, T.; Lehto, S.; Kallioniemi, A.; Schleutker, J.; Butzow, R.; Blomqvist, C.; et al. Recurrent moderate-risk mutations in Finnish breast and ovarian cancer patients. *Int J Cancer* **2019**, *145*, 2692-2700, doi:10.1002/ijc.32309.
2. Vehmanen, P.; Friedman, L.S.; Eerola, H.; McClure, M.; Ward, B.; Sarantaus, L.; Kainu, T.; Syrjakoski, K.; Pyrhonen, S.; Kallioniemi, O.P.; et al. Low proportion of BRCA1 and BRCA2 mutations in Finnish breast cancer families: evidence for additional susceptibility genes. *Hum Mol Genet* **1997**, *6*, 2309-2315, doi:10.1093/hmg/6.13.2309.
3. Syrjakoski, K.; Vahteristo, P.; Eerola, H.; Tamminen, A.; Kivinummi, K.; Sarantaus, L.; Holli, K.; Blomqvist, C.; Kallioniemi, O.P.; Kainu, T.; et al. Population-based study of BRCA1 and BRCA2 mutations in 1035 unselected Finnish breast cancer patients. *J Natl Cancer Inst* **2000**, *92*, 1529-1531, doi:10.1093/jnci/92.18.1529.
4. Sarantaus, L.; Huusko, P.; Eerola, H.; Launonen, V.; Vehmanen, P.; Rapakko, K.; Gillanders, E.; Syrjakoski, K.; Kainu, T.; Vahteristo, P.; et al. Multiple founder effects and geographical clustering of BRCA1 and BRCA2 families in Finland. *Eur J Hum Genet* **2000**, *8*, 757-763, doi:10.1038/sj.ejhg.5200529.
5. Pallonen, T.A.; Lempiainen, S.M.M.; Joutsiniemi, T.K.; Aaltonen, R.I.; Pohjola, P.E.; Kankuri-Tammilehto, M.K. Genetic, clinic and histopathologic characterization of BRCA-associated hereditary breast and ovarian cancer in southwestern Finland. *Sci Rep* **2022**, *12*, 6704, doi:10.1038/s41598-022-10519-y.
6. Huusko, P.; Paakkonen, K.; Launonen, V.; Poyhonen, M.; Blanco, G.; Kauppila, A.; Puistola, U.; Kiviniemi, H.; Kujala, M.; Leisti, J.; et al. Evidence of founder mutations in Finnish BRCA1 and BRCA2 families. *Am J Hum Genet* **1998**, *62*, 1544-1548, doi:10.1086/301880.
7. Hartikainen, J.M.; Kataja, V.; Pirskanen, M.; Arffman, A.; Ristonmaa, U.; Vahteristo, P.; Ryynanen, M.; Heinonen, S.; Kosma, V.M.; Mannermaa, A. Screening for BRCA1 and BRCA2 mutations in Eastern Finnish breast/ovarian cancer families. *Clin Genet* **2007**, *72*, 311-320, doi:10.1111/j.1399-0004.2007.00866.x.
8. Pelttari, L.M.; Shimelis, H.; Toiminen, H.; Kvist, A.; Torngren, T.; Borg, A.; Blomqvist, C.; Butzow, R.; Couch, F.; Aittomaki, K.; et al. Gene-panel testing of breast and ovarian cancer patients identifies a recurrent RAD51C duplication. *Clin Genet* **2018**, *93*, 595-602, doi:10.1111/cge.13123.
9. Barkardottir, R.B.; Sarantaus, L.; Arason, A.; Vehmanen, P.; Bendahl, P.O.; Kainu, T.; Syrjakoski, K.; Krahe, R.; Huusko, P.; Pyrhonen, S.; et al. Haplotype analysis in Icelandic and Finnish BRCA2 999del5 breast cancer families. *Eur J Hum Genet* **2001**, *9*, 773-779, doi:10.1038/sj.ejhg.5200717.
